# Existential anxiety, psychological flexibility, and deep resilience to climate crises

**DOI:** 10.3389/fpsyg.2025.1628080

**Published:** 2025-10-23

**Authors:** Carl F. Weems, Cristina Poleacovsch, Scott Feinstein, Marcus Nartey

**Affiliations:** ^1^Department of Human Development and Family Studies, Iowa State University, Ames, IA, United States; ^2^Department of Civil, Construction, and Environmental Engineering, Iowa State University, Ames, IA, United States; ^3^Department of Political Science, Iowa State University, Ames, IA, United States

**Keywords:** deep resilience, climate, crisis, psychological flexibility, existential anxiety

## Abstract

Concerns and uncertainty about the viability of continued existence on the planet are central to the urgency of the climate crisis and can be contextualized within the philosophical idea of existential anxiety. The goal of this conceptual analysis is to outline how existential anxiety and psychological flexibility may help in understanding reactions to climate crises and thereby facilitate what has been termed “deep resilience.” This paper offers a definition of deep resilience that may be operationalized and measured, incorporating the concepts of existential anxiety and psychological flexibility. Simply put, deep resilience is multilayered, multiscale flexibility and adaptation to existential threat. Broadly, deep resilience involves flexible adaptation at the individual, family, community, societal, and geographical scales in response to existential threats to individuals, families, communities, societies, geographical regions, and the planet itself. The paper explores the conceptual and empirical foundations for these concepts as a potential basis for defining, acting toward, and actualizing deep resilience. Examples from the empirical literature spanning psychological, climate-related, and political domains are discussed, with avenues for future research and policy noted.

## Introduction

Existential anxiety involves apprehension about life and death—that is apprehension and uncertainty about existence. This conceptual analysis examines how these concerns relate to the climate crisis and the development of deep resilience in response. Concerns about the viability of continued productive existence on parts of the planet and even Earth itself are central to the urgency of the climate crisis. Chungyalpa et al. (2025) write “The Anthropocene refers to an era of deep uncertainty and potential collapse, which demands that we prepare for this reality immediately and at all scales across the individual, societal, business, governance, and global levels and invest in building inner, community, and ecological resilience simultaneously and equally” (Chungyalpa et al., 2025, conclusion section, paragraph 1).

Concerns about the climate crisis or particular crises have been examined as eco-anxiety and climate anxiety (Lammel, 2025; Wray, 2023). We use the phrase “the climate crisis” (singular) when referring to the overarching, ongoing global phenomenon of climate change and its widespread effects. There are, however, many specific components (climate crises, plural), the primary singular term “crisis” emphasizes a single, complex, and systemic problem requiring urgent action. Action to address the crisis involves individual and collective action (Lammel, 2025; Lawrence et al., 2024) toward deep resilience on specific crises as well as the broader crisis. For example, Lammel (2025) has noted that the crisis is at least threefold involving climate change, biodiversity loss, and pollution. This global “polycrisis” has been defined as a causal entanglement of crises across multiple global systems—such as climate, economy, health, and geopolitics that fundamentally damage humanity’s future well-being (see Lawrence et al., 2024; Richardson, 2025).

While eco-anxiety, climate anxiety and existential anxiety are distinctive (Oksala, 2023), uncertainty is central to all of these concepts (key concepts are defined in Table 1). Climate anxiety is specific to concerns about climate change and environmental collapse, while existential anxiety is a broader concept—defined more fully later in the paper. Climate anxiety often contains an existential component—because climate change threatens not just ecosystems but the future of humanity, amplifying questions about survival and meaning. We would argue that while eco-anxiety or climate anxiety are fundamentally important concepts, the broader concept of existential anxiety is particularly useful in moving toward deep resilience approaches. This broader concept, with deep historical and conceptual roots may be harnessed to promote deep resilience regardless of whether particular individuals or groups of people are concerned directly about the broader environment or climate. The goal of this conceptual analysis is to outline how both existential anxiety and psychological flexibility (i.e., the ability to shift mindsets and behavioral response sets) may help in defining and understanding human reactions to climate crises and thereby facilitating what has been termed “deep resilience.”

**Table 1:** Key concepts.

Term	Definition
Existential anxiety	A broad concept with a long philosophical basis that involves apprehension about the ultimate meaning in life and death—apprehension and uncertainty about existence, guilt and morality (Tillich, 1952; Weems et al., 2004)
Eco and climate anxiety	More specific concepts than those of existential anxiety. Eco-anxiety is the broader of the two terms and is anxiety about a range of environmental issues, including biodiversity loss/deforestation, pollution, and climate change. Climate anxiety is related to anxiety/fear tied to concerns about climate change and its consequences—these may be; however, extreme weather, rising sea levels, biodiversity loss. Both concepts have existential dimensions (Hickman et al., 2021; Wray, 2023)
Ecological systems theory of human development	Tied to Uri Bronfenbrenner’s theory that describes the influences on human development by multiple concentric layers of environmental systems and also time. The *individual* is situated in the *microsystem*, which is the close environment—family, school, and peers. The *mesosystem*, which represents the interconnections among microsystems (e.g., how family life affects school experiences). The *exosystem* includes broader settings that indirectly influence the individual, like a parent’s workplace or community services. The *macrosystem* encompasses cultural values, laws, and societal norms that shape all other systems. The *chronosystem* adds the dimension of time, capturing change, transitions and historical events. In disaster research, this is often simplified to three main levels Micro-level- individual and immediate settings such as family (original individual and microsystem); Meso/Exo-level-community and institutional contexts (mesosystem and exosystem); Macro-level- Societal and cultural structures, including time (macrosystem and chronosystem; Bronfenbrenner, 1979; Weems et al., 2023; Weems, 2019)
Psychological flexibility	The capacity to adjust to different situational demands, shift perspectives or behaviors when current strategies hinder personal or social well-being, maintain equilibrium across key areas of life, and act with awareness, openness, and commitment to behaviors aligned with core values (Kashdan and Rottenberg, 2010).
Resilience	The process of adapting well in the face of adversity, trauma, or significant stress, maintaining or returning to healthy functioning over time. The ability of is any system (biological, psychological, social) that interacts with its environment and evolves over time an individual/family, or community respond, adapt change successfully to challenges that threaten function, survival, or development (Masten, 2025; Weems and Graham, 2014)

### Defining deep resilience

Muller et al. (2024) state: “Deep resilience is achieved through a multi-actor, multi-layered, and multi-dimensional approach. This approach combines resources, capabilities, and information to leverage the strengths of individuals, public and private organizations, and systemic governance. By doing so, it enables effective preparation for, response to, and recovery from disruptions” (Muller et al., 2024; p. 5) Similarly, Chungyalpa et al. (2025) in discussing deep resilience point to inner or personal individual aspects, community or social aspects and planetary aspects of resilience again emphasizing a multi-layered multidimensional approach. However, we could find no clear definitions of this concept in the published literature. This paper proposes such a definition building on prior conceptualizations of resilience, existential anxiety, psychological flexibility, and systems theory.

Our work addressing climate crises is built on prior efforts focused on disaster response, preparedness, and recovery from traumatic and adverse experiences, and emphasizes the multidimensional nature of resilience (Weems et al., 2021, 2023). We propose a definition of deep resilience that may be operationalized and measured, which draws on the ideas noted in Chungyalpa et al. (2025) and Muller et al. (2024) as well as existing definitions of resilience (see Table 1; Masten, 2025; Weems and Graham, 2014). Our definition expands these to include existential concerns, psychological flexibility, and the multiple levels of analysis in systems theory. Briefly, deep resilience can be defined as multilayered, multiscale flexibility and adaptation to existential threat. The broader definition of deep resilience involves flexible adaptation at multiple levels such as the individual, family, community, society, and geographical scales in response to existential threats to individuals, families, communities, societies, geographical regions, and the planet.

The remainder of this conceptual analysis provides an initial outline of the theoretical and empirical basis for this definition. As a novel argument, with inchoate evidence, we do not have the same goal as in a systematic review but to simply present these ideas for further evaluation as to their utility using narrative review.

### Background framework

Our previous work (Weems, 2019; Weems et al., 2021, 2023) reflects the role of multiple levels of influence on resilience and response to climate-related crises. This work has drawn from the ecological systems theory of Uri Bronfenbrenner (Bronfenbrenner, 1979; Shelton, 2025). This model describes the influences on human development by multiple concentric layers of context termed “environmental systems”^1^ and also time (see Table 1 for a full definition). In our definition of deep resilience, at the broadest level, resilience is supported by protective landscapes (environments such as wetlands) and overarching governmental policies. These include geographic features, national infrastructure, cultural values, regional or national norms as well as the historical time frames or zeitgeists. While actions at this scale have the potential for widespread impact, their implementation depends on engagement from various communities. Communities—ranging from municipalities and counties to entire states—function within (are nested in) these larger societal and geographic frameworks. Each community responds to crises in unique ways, drawing on resources such as social capital, public trust, local governance, and community-driven initiatives like recycling programs or environmental clean-up efforts.

Within communities are nested organizations. At this organizational level of analysis, the focus is on the governance decision-making processes such as laws and policies, as well as engineering and community planning. In other words, various organizations choose how and to what extent to carry out broader policy suggestions, whether to evade or follow broader laws designed to mitigate and prevent climate impacts such as policy work targets and other climate actions. For example, businesses may choose to have specific job responsibilities related to sustainability and/or conservation, or to simply do the minimum to achieve compliance with relevant laws and regulations.

Families and individuals are the smallest units of analysis and are nested within each of the larger contexts. Families and individuals are also prime movers of actions at the upper levels of analysis. Individuals function within families and other small units like friendship groups. At this level, fostering deep resilience involves examining factors within the individual and within families that promote resilience to climate crises. For example, at the individual level, direct exposure to threat is associated with a host of health and mental health issues across development. For example, exposure to Traumatic and Adverse Childhood Experiences (TRACEs; Weems et al., 2021) brought on by climate-related disasters are associated with differential health and mental health outcomes as well as differential physical development such as brain development. TRACEs differentially impact individuals and families. These differential effects may lead to both resilient and negative outcomes following the same or similar risk.

Those with higher risk for directly experiencing climate-related impacts, have a theoretically more salient understanding of climate impacts (Guagnano and Markee, 1995). However, geographic region may not directly predict climate concerns, it is more likely these interact with other variables to influence environmental concern (Guagnano and Markee, 1995; Hoffmann et al., 2022; Lewis et al., 2019). Thus, in addition to cumulative effects across levels of analysis, our previous work predicts important interdependencies, and individual differences (such as in general existential concerns and psychological flexibility). Specifically, that communities, organizations, families, and individuals are nested (see Figure 1A in Weems et al., 2023). The nesting suggests that resilience at one level can influence and reinforce resilience at other levels in complex ways. This layered complexity helps identify both vulnerabilities and sources of resilience in the face of existential crises. The concept of existential anxiety is not new. However, existential threat to each of the layers of deep resilience as defined above makes this old concept increasingly relevant in contemporary climate contexts.

## Existential anxiety

Existential anxiety as a concept has been the subject of theoretical and philosophical writing for close to 200 years (e.g., Camus, 1959; Frankl, 1963; Kierkegaard, 1954a; Kierkegaard, 1954b; Nietzsche, 1878/1996; Sartre, 1957; Tillich, 1952; Yalom, 1975) if not earlier (see Ecclesiastes 1, New International Version Bible, 2011). While there are many existential perspectives that relate to climate crises (see Vaškovic, 2023), our work on existential anxiety (Weems et al., 2004; Berman et al., 2006) is based primarily on the writings of Paul Tillich (1952) who outlined several facets of existential threat.

### Tillich’s existential domains

Tillich (1952) conceptualizes existential anxiety as arising from three interconnected domains. The first is the anxiety of fate and death, which reflects the fear of nonexistence and the personal uncertainty tied to one’s destiny. The second domain involves emptiness and meaninglessness—anxiety that stems from the fear that life lacks a deeper purpose or “ultimate concern.” The third domain is guilt and condemnation, which relates to the fear of moral failure and the threat to one’s ethical self-concept.

Each of these domains, according to Tillich (1952), includes both relative and absolute dimensions. For instance, anxiety about fate is a relative concern tied to the unpredictability of life events, while the fear of death represents an absolute anxiety that underlies all others. Similarly, emptiness reflects a relative fear that one’s beliefs have lost their significance, whereas meaninglessness is the absolute fear that life itself holds no meaning. In the final domain, guilt is a relative anxiety about failing to meet personal standards, while condemnation is the absolute fear of falling short of universal moral expectations. Together, these domains and their subcomponents form a comprehensive framework for understanding existential anxiety.

The idea presented in this conceptual analysis is that each of these domains can help to understand avenues to resilience or non-resilience in each of the interconnected domains needed for deep resilience. In other words, Tillich (1952) conceptual view of existential anxiety is useful to show that different facets of existential concerns may facilitate or hinder deep resilience.

Data on the role of climate anxiety and eco-anxiety is emerging as a potentially important mental health concern (Hickman et al., 2021; Lammel, 2025; Wray, 2023). Lammel (2025) has pointed out that these concerns may affect individuals directly through extreme weather events, indirectly through consequences like famine, and also vicariously through media exposure (see also Thapa et al., 2025). As with these specific concerns, the broader concerns of existential anxiety may become particularly salient in the aftermath of climate-related traumatic stress (Scott and Weems, 2013; Weems et al., 2016). Individuals forced to confront their own mortality may become preoccupied with questions about the meaning of life and death (Pyszczynski et al., 1999). Scott and Weems (2013) found that existential concerns based on Tillich (1952) framework and measured by the Existential Anxiety Questionnaire (Weems et al., 2004) were widespread among adults affected by Hurricane Katrina. Their study also revealed a strong positive association between PTSD symptoms and all six of Tillich’s existential domains. Compared to individuals who had not experienced the disaster, those exposed reported significantly more concerns related to death, fate, emptiness, condemnation, and guilt, and were more likely to express concerns across multiple domains.

Building on this, Weems et al. (2016) explored existential anxiety among 325 adolescents living in the Greater New Orleans area who had been exposed to Hurricanes Katrina and Gustav. Their findings mirrored those of Scott and Weems, showing that each of Tillich (1952) six existential domains was commonly endorsed. Specifically, high percentages of adolescents reported concerns about fate (87.5%), death (66.7%), guilt (79.1%), emptiness (79.8%), and condemnation (81.1%), with slightly fewer expressing concerns about meaning (32.5%). When compared to a non-exposed adolescent group, those who had experienced the disasters reported significantly higher levels of existential anxiety in all domains except meaninglessness. Importantly, existential anxiety in this group was associated with elevated symptoms of PTSD and depression. Moreover, the level of disaster exposure influenced the strength of this relationship—those with higher exposure and greater existential concerns experienced the most severe mental health symptoms. These findings underscore the relevance of existential concerns in youth affected by disasters and suggest that such exposure may intensify normal existential anxieties, increasing their impact on psychological well-being.

Two important related points: First, these data come entirely from individuals and so research is needed to move this to broader levels implied by systems theory. Second is the role of balance and flexibility in understanding the implications of existential concerns. While high existential anxiety is associated with higher symptoms of anxiety and depression and exposure to climate-related events may exacerbate existential anxiety and its linkages to psychological problems, some level of existential anxiety is not inherently positive or negative. A point of the philosophers noted above is that existential anxiety is core to the human condition. A main point of this conceptual analysis is that balancing existential concerns through psychological flexibility is critical for developing deep resilience.

## Psychological flexibility

Psychological flexibility can be defined as the capacity to “recognize and adapt to various situational demands; shift mindsets or behavioral repertoires when these strategies compromise personal or social functioning; maintain balance among important life domains; and be aware, open, and committed to behaviors that are congruent with deeply held values” (Kashdan and Rottenberg, 2010). Writers on eco-anxiety have noted the potential importance of psychological traits and states generally in shaping responses to climate change (Boivin et al., 2025).

While the empirical literature connecting these is scant, psychological inflexibility has been shown to exacerbate the association between climate change concerns and climate anxiety (Feather and Williams, 2022). Specifically, Feather and Williams (2022) reported psychological inflexibility as a moderator of the association between concern about climate change and climate-related distress (*n* = 771) specifically that the link between climate change concerns and climate-related distress was stronger among participants who reported higher psychological inflexibility. Psychological inflexibility refers to a tendency for behavior to be overly governed by negative emotions such as anger, fear, or anxiety and rigid thoughts or beliefs, especially those that are ideologically fixed rather than by action toward goals based on personal values (Bond et al., 2011; Hayes et al., 2002; Zarling et al., 2023).

The concepts of psychological flexibility and inflexibility grew from Acceptance and Commitment Therapy (ACT). ACT therapists often target experiential avoidance with their clients. Experiential avoidance is a specific form of psychological inflexibility. Experiential avoidance involves efforts to suppress or control internal experiences (Hayes et al., 1996, 1999). However, rather than attempting to eliminate or suppress cognitive errors, negative beliefs or negative emotions, ACT focuses on cultivating psychological flexibility by fostering commitment to behavioral goals and skills training. The goal is not to eliminate problematic thoughts or emotions, but to reduce their influence over desired behavior. ACT and the related concepts of flexibility have broad application with research showing its effective for the treatment of depression, anxiety and aggression (Hayes et al., 1996, 1999, 2002; Zarling and Russell, 2022; Zarling et al., 2015, 2023, 2025).

In summary, psychological flexibility, as cultivated through ACT techniques, enables individuals to pursue values-driven actions. This capacity to adapt and remain engaged is foundational to deep resilience, allowing individuals and communities to respond constructively to climate-related threats.

## Conceptual integration of existential anxiety and psychological flexibility toward deep resilience

Table 2 outlines three main levels of deep resilience in the definition above: The broadest level are human societies and broad geography,^2^ followed by an intermediate scale of community and region, and then the smallest level—individuals and families. These levels should not be thought of as bounded and inseparable. Ecological systems theory of human development (Shelton, 2025) identifies multiple levels all interacting. The individual is the basis and is situated in the microsystem, which is the individual’s close environment (i.e., family, school, and peers). This smallest level is classed the “Micro-level.” The second level consists of the mesosystem, which represents the interconnections among microsystems (e.g., how family life affects school experiences) and the exosystem includes broader settings that indirectly influence the individual, like a parent’s workplace or community services. This level is called the “Meso-level.” The final broadest level includes the macrosystem which encompasses cultural values, laws, and societal norms that shape all other systems and the chronosystem which adds the dimension of time, capturing change, transitions and historical events. This third level is often called the “Macro-level” (Bronfenbrenner, 1979; Weems et al., 2023; Weems, 2019).

**Table 2:** Existential concerns and their conceptual relation to risk and resilience.

Levels	Existential belief dimensions toward deep resilience		
Society and geographical scale 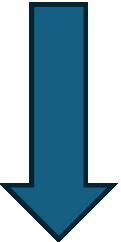	Concerns Toward Risk ← Valence → Toward Resilience		
	Fate and Death	The death of the planet is inevitable because there is nothing we can do. Our culture is not anxious about the planet’s fate because society has normalized ecological decline.	Considering the death of the planet motivates my country to protect it. The fate of the planet depends on our shared societal choices and values.
	Emptiness and Meaning	Life on this planet has no meaning. Human actions to protect the earth are empty.	Our planet provides meaning to human existence. Thinking about the planet’s future motivates us to act in the present, even if those actions may ultimately feel empty or meaningless within the vastness of time.
	Guilt and Condemnation	We feel no guilt about climate change on earth. Life on earth is ultimately condemned.	Humans are at least partially guilty as responsible for climate change on earth. Life on earth is not ultimately condemned if humans act.
Community and regional scale 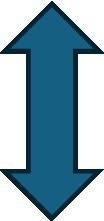	Toward Risk ← Valence → Toward Resilience		
	Fate and Death	The death of (e.g., forests, wetlands, and species) in my region is inevitable—there is nothing we can do. We are not anxious about fate of my community—We are resigned to destiny.	The thought of forests, wetlands, and species disappearing from my region compels us to act in their defense. The fate of the community is in our hands to change.
	Emptiness and Meaning	Our community/region does not provide meaning. Community actions to protect the environment are empty.	Protecting the environment of my community provides meaning. Our community actions to protect the environment are not empty.
	Guilt and Condemnation	We feel no guilt about climate change in my country/region/community. The community is condemned to climate crises.	We address guilt about climate crises in my region by acting in my community. Our region is not condemned to climate crises if we act.
The individual and families 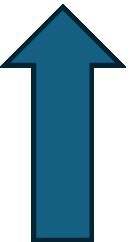	Toward Risk ← Valence → Toward Resilience		
	Fate and Death	I get anxious thinking about getting old and dying. I think about my fate and it causes me to feel anxious because there is nothing I can do.	I am not anxious about death because I feel my life has meaning through the legacy I’ve created with my family. I am not anxious about my fate because my actions will help.
	Emptiness and Meaning	I am anxious because my life is empty. Our family has no real meaning or purpose.	Life has meaning. I act to create meaning. As a family, we do not feel a sense of emptiness—we find purpose in each other and working for our shared future.
	Guilt and Condemnation	Our family feels anxious because of guilt passed down through generations (Our family acts to address the guilt passed down through generations of neglecting the environment.) I feel anxious because I feel condemned (I act to prevent a feeling condemnation from neglecting the environment).	We are not worried about guilt because we have learned to forgive within our family and work to create positive change. I do not feel anxious about being condemned, because I act to effect change.

The examples in the table are just a few examples of a myriad of variations, for example at the broad level the fate and death items have the sentiments that lead to action while the emptiness and meaning sentiments are the effects of inaction. While the broader levels are collective—the I statements reflect that individuals hold the beliefs. The “we” and “our” to indicate a collective stance that may be expressed in societal sentiments or community norms. The statements of existential positions toward risk and resilience also attempt to have illustrative examples crossing individuals and families, regions and communities, and also broad societies and time. Importantly, what may be associated with resilience for an individual may not necessarily be toward deep resilience for broader levels. Similarly, beliefs associated with risk for an individual may motivate action toward deep resilience for broader levels. See the parenthetical examples at the individual and family level for guilt and condemnation - where individual or family guilt (risk) could motivate broader action (resilience). See text for additional discussion of this point.

Table 2 includes examples of how different perspectives on each of the existential concerns (fate/death, guilt/condemnation, emptiness/ meaninglessness) might be related to increased threat and also how the concerns may also help to foster deep resilience. For example, at the society and geographical scale, existential concerns centering on fate and death such as thinking that “the death of the planet is inevitable because there is nothing we can do about it,” or “Our country is not anxious about the fate of the planet because we are resigned to destiny”—might be reassuring to some in terms of uncertainty—but is unlikely to foster deep resilience and may indeed increase the risk humans pose. However, if thinking about the death of the planet causes societies to take action to protect it or believing that the fate of the planet is in human society’s hands to change is fundamentally movement toward deep resilience. Additional examples, for this broadest dimension of deep resilience to existential risk for guilt/condemnation, emptiness/meaninglessness are in Table 2. To the extent that the broadest levels of government and society embrace existential perspectives toward deep resilience—human society will be moving toward better managing climate crises. These broadest perspectives on the existential threat, however, come from the communities and regions nested in these broadest contexts.

At the community and regional levels, for example, in terms of the existential concerns of emptiness and meaninglessness thinking that the community/region does not provide meaning or that community actions to protect the environment are empty is likely to increase risk. However, thinking that protecting the environment of my community provides meaning and believing that community actions to protect the environment are not empty is likely to foster resilience. Similarly, in terms of guilt and condemnation, feeling no guilt about climate change in one’s country/region/community or believing the community is condemned no matter what conceptually increases risk. In contrast, addressing guilt about climate crises by acting in the community or believing the region is not condemned to destruction if the community acts, is “toward deep resilience.” As final examples, in terms of fate and death, believing that the death of (e.g., forests, wetlands, and species) in my region is inevitable or not having any anxiety about the fate of the community or region conceptually increases risk. But if thinking about the death of (e.g., forests, wetlands, species) in my region causes community action to protect it and believing the fate of my community is in our hands to change moves toward deep resilience.

All of these broader concerns derive from the individuals nested within communities and examples of existential concerns for individuals and families as outlined in Table 2. The individual has been the focus of much of this literature and this is where action toward deep resilience starts in our individual belief systems but also in the flexibility of our belief systems to evolve and change with information and a balance across the individual’s existential concerns and broader levels. The examples in Table 2 at the individual and family level are not set to climate change examples but to the core concept of individual existential concerns as felt by an individual regardless of the reason. As comparison across the levels in Table 2 helps to show, at the individual level, resignation to fate and death, low guilt and condemnation and an inherent or created sense of meaning in life is *protective*—*toward individual resilience*. This is the opposite at the broader levels where *concerns about guilt and lack of a resignation to fate should conceptually motivate action toward deep resilience*.

It is worth emphasizing this point, As Budziszewska and Jonsson (2021) have pointed out “Although environmental worries are legitimate, they sometimes cause severe anxiety and distress so aggravated as to be discussed within the framework of psychotherapy.” Thus, at an individual level, the individual’s personal experience is somewhat opposite to the resilience building that existential concerns may foster at the broader levels. For example, guilt concerns around human pollution and its environmental impact became increasingly salient in the 1970s leading to major policy reforms, including the establishment of the US Environmental Protection Agency, the Clean Water Act and the Endangered Species Act (Weart, 2008).

Similarly, developed countries’ outsized impact on climate change has contributed to guilt concerns that have been linked to successful global environmental agreements, like the Kyoto Protocol and the Paris Climate Agreement (Okereke, 2010). One specific example of success was as at the Copenhagen climate summit in 2009 in which developed countries pledged “new and additional resources, including forestry and investments…approaching USD 30 billion for the period 2010–2012…and reaching USD $100 billion dollars a year by 2020 to address the needs of developing countries.” (UNFCCC, 2010). This difference in how existential concerns can motivate both toward resilience and toward fate depending on the target of action individual versus outward highlights the importance of flexibility and balancing how such concepts are employed to promote deep resilience.

As another example, resignation to fate and death could make an individual more resilient in the sense that they accept risks and worry less and are more individually “healthy” however, what is good for the individual mental health is not necessarily good for the community or nation or planet- and ultimately the individual. Just because one does not think they will be impacted and do not experience the trauma of worrying about risks or struggling to do something about risks—this does not mean whatever risk or threat is out there does not impact them. This balancing act of the potential differential impact of existential concerns at the individual and family levels and simply balancing the level of existential concerns highlights why psychological flexibility is also a key component.

### Flexibility and balance as keys to existential concerns toward deep resilience

Fostering flexibility in thinking about existential concerns is important to deep resilience. For example, if global temperature rise above 2 degrees Celsius—does that mean we should switch from existential action to resignation—no it does not. However, it is often hard for individuals to understand and accept change and understand the ever—changing perspectives that new data give to scientific conclusions. In other words, it is often difficult for the average person to appreciate that scientific conclusions are typically tentative (Compton et al., 2021; Lewis et al., 2023; Miller, 2022).

A good example of this is the United Nations on the Paris Climate Agreement which indicates that the agreement “helps us to avoid locking in a level of ambition that would make the well below 2 degrees goal improbable. Countries will have an opportunity to review their collective effort against the global goals prior to formally submitting their national contributions to the new agreement. This exercise will be repeated every 5 years. We have an agreement and we have a chance now to reach our goal. We could not say that without an agreement… …We did not expect to leave Paris with commitments to reach that goal, but rather, with a process that will get us there” (United Nations, 2016). People and policymakers often prefer a more dichotomous reality where there is yes and no, true and false, and are less comfortable with gray zones or “approximations” toward truth. Shifting perspectives and mindsets in response to new data is theoretically critical to fostering deep resilience in the conceptual perspective outlined here. The techniques in ACT may be an avenue for building psychological flexibility and help individuals, families, communities and societies engage in values-based actions toward deep resilience to climate-related existential threats.

This idea of balancing the level of existential concern can be illustrated by the classic concept that performance improves with increased arousal—but only up to a point. After that, too much arousal can actually impair performance. This concept is known as the Yerkes-Dodson Law and is often illustrated as an inverted U-shaped curve with low arousal and low performance, moderate arousal and optimal performance (i.e., and ideal balance of focus and energy), but diminishing gains at high arousal. This concept has evolved and is also referred to as optimal arousal theory (see Hanoch and Vitouch, 2004). Not being at all anxious about death or your fate in the face of a hurricane is likely to result in not acting- and likely leading to a bad outcome. So being overwhelmed with fright may similarly lead to poor decisions.

## Discussion

This conceptual analysis showed how work to address climate crises might be facilitated by integrating the philosophical idea of existential anxiety and psychological flexibility. Building on previous work, we proposed a definition of deep resilience that includes the concepts of existential anxiety and psychological flexibility that may help to operationalize the idea as well as assess public sentiment, foster policy and other actions toward deep resilience. For example, understanding community existential concerns around climate issues and their psychological flexibility in responding to information on climate impacts and actions may help facilitate deep resilience. Human existential concerns about the climate are multilayered, and require flexible adaptation at the individual, family, community, society, and geographical scales in response to the existential threats to individuals, families, communities, societies, geographical regions and the planet itself.

### Illustration from work in Alaska native and Puerto Rican communities

Data from our work in Alaska Native communities (Cantrell et al., 2025; Nartey et al., 2024, 2025) and Puerto Rico (Ballesteros et al., 2025; Nelson et al., 2023, 2025) illustrates the conceptual analysis provided herein. As part of the Alaska project, interviews with community members (*N* = 53) in the Norton Sound Health Region (Nartey et al., 2024) focused on perceptions of water infrastructure and drinking water in the community, allowing respondents to freely express their concerns. The data reveal that the effects of climate change are palpable in the Arctic region, particularly in remote Alaskan communities. Infrastructural decay, erosion, and seasonal water instability are some of the climate change effects common in many Alaska Native communities. These challenges are particularly pronounced due to the importance of water in the culture but also historic disinvestment and systemic underfunding of water infrastructure projects. The result is grave water accessibility challenges as well as deep community-wide mistrust in municipally treated water (Eichelberger, 2019). This context reveals how existential concerns about safety, control, and the future are not abstract in these communities. They are the lived experiences and reality that influence individual daily decisions but also motivation for collective action.

*Individual-Level Responses and Existential Anxiety:* On the individual level, many community members express low trust in treated water due to previous experiences with contamination, discoloration of water, or illness (Nartey et al., 2024). This mistrust reflects existential anxiety, particularly concerns about fate/death (“Is this safe?”) and emptiness/meaninglessness (“Why cannot we even trust our water?”). Even natural sources of water such as rivers, snow, and spring water, which were historically trusted and central to Alaska Native way of life are beginning to face skepticism from individuals due to concerns about their safety in the light of climate change. The erosion of the “meaning” in community life supplied by “traditional” water access and integration into community life and the threat modern water systems impose deepens individual-level existential anxiety. Understanding how these concerns and beliefs not only challenge the core frameworks through which people make sense of their environment, but also their potential willingness to act collectively.

As with many other societies, the individual-level existential anxieties in Alaska Native communities accumulate and reflect in family-level responses. Indeed, similar themes from qualitative analysis can be seen in our work in Puerto Rico following the damage related to Hurricane Maria and the individual/family intersections with organizational, community, governmental tap water infrastructure (see Nelson et al., 2023, 2025) and reconstruction efforts in the design decisions of informal rebuilding efforts (see Ballesteros et al., 2025).

An adaptive response noticeable in many households is the practice of hauling water from rivers, rain, or snow, which although face some level of skepticism, are more trusted than treated water. While this is sometimes done out of necessity (boil water alerts or water outages preventing access to treated water), mistrust is another significant reason for the reliance on these alternative sources of water. Other households, however, continue using the treated water even if they mistrust it, out of fatigue, necessity, or resignation. This contrast of responses shows that existential anxiety does not always result in productive adaptation; it can also lead to disengagement (Nartey et al., 2024, 2025).

*Community-Level Adaptations and Challenges:* At the community level, responses to water insecurity tend to focus on immediate mitigation rather than long-term climate adaptation. Tribal councils and local governments have taken practical steps such as providing bottled water, encouraging conservation, and applying for external funding to improve or replace aging infrastructure (Nartey et al., 2024, 2025). While these efforts reflect a degree of institutional engagement and resourcefulness, they are largely reactive, addressing symptoms rather than causes. Climate change itself is not always openly named as the driver behind these disruptions, and the broader attitude within the community often reflects a sense of apathy or emotional distance from a climate crisis. Such a collective posture may stem from a long history of broken promises, marginalization, and fatigue from navigating chronic infrastructure challenges (Walters et al., 2010; Wilson and Inkster, 2018). As a result, even well-meaning initiatives can be met with skepticism, and momentum toward deeper systemic change is limited. This illustrates a form of community-level existential anxiety which is not dramatic or outspoken, but quietly embedded in a perceived lack of control, resignation, and disconnection from larger-scale climate action narratives.

*Macro level:* Broader state and national policies further complicate this picture. As Cantrell et al. (2025) have pointed out drinking water systems in Alaska Native communities differ significantly from those in the contiguous US, with limited documentation and unclear governance structures. Fragmented, missing, and inaccessible data further complicate efforts to understand and improve these systems, hindering progress toward sustainable and efficient water infrastructure in remote areas. Their findings highlight found challenges in the availability, accessibility, adequacy, and utilization of water systems data. Addressing these climate, policy, pollution and habitat related problems involves the technical, financial, and operational aspects of these systems, but also the critical roles of the organizations involved in supporting community water management from the local to the federal government.

These intersections across levels (individuals, families, community infrastructure, and state policy) can also be illustrated in post disaster recovery in Puerto Rico. Nelson et al. (2023, 2025) results suggest that even after significant post-Hurricane Maria improvements in community infrastructure and state policy, many residents (individuals and families) still avoid tap water—not because of current quality, but because of persistent memories of past failures. This “knowledge–behavior gap” illustrates the psychological processes described in Table 2. Individuals may reinterpret positively or never forget an existential threat, but their perceptions of communities and policy may not align with improved safety and reliability. Theoretically, deep resilience to crises is not just about rebuilding better systems; it is about rebuilding trust at each of the levels of analysis in the definition we proposed. Transparent communication, community engagement, and acknowledgment of lived experiences are as critical as engineering fixes. In both mental health and infrastructure, resilience depends on addressing the narratives people carry forward about themselves, their families, communities and larger socio-political context.

This multi-layered example from a community whose way of life intersects with the natural environment highlights how existential anxiety and psychological flexibility manifest in uneven ways across scales, as illustrated in Table 2. Framing these as problems as existential threats but also solvable by flexible responses to shared goals may facilitate action—regardless of the nature of the one’s belief about impact of climate change. However, not directly connecting these experiences to “climate change” publicly may be strategic in encouraging skeptics or deniers to engage with behavior change and policy that is needed for deep resilience. Thereby serving an important purpose—even if it does increase the psychological distance between an experience and its cause. This nuance may be one of the biggest challenges to this perspective. This balancing the level of existential concern that is optimally motivating and balancing the individual’s beliefs about what is good for them with what is good more broadly is more easily said than accomplished.

## Conclusion

Ultimately, there is a need to transcend opposing existential rhetoric to build deep resilience. Identifying shared existential goals and action toward those goals may be fostered by recognizing the broadest existential concerns of humans, families, communities and societies. For example, the same existential concern “This is destroying our way of life forever—a way of life that we have cherished for generations” can be held by both those who believe in climate change have eco-anxiety and those who do not. Research on ACT has shown that combating cognitive errors such as catastrophizing with opposing rhetoric is often futile. The analogy is that combating commercial and industrial economic interests that harm the environment and climate with opposing existential rhetoric is likely futile as well. Both sides of the debate use this rhetoric. The International Court of Justice calling global warming an “urgent and existential threat” (Zraick, 2024) and the same rhetoric being used for the loss of fossil fuel and manufacturing industries (see Kinniburgh, 2023; Kota and Mahoney, 2020). By recognizing shared existential concerns action toward deep resilience may succeed.

In conclusion, we defined deep resilience as multilayered, multiscale flexibility and adaptation to existential threat. Recognizing threats not only to the survival of the planet and individuals or cultures, but also to deeper aspects like personal meaning, fulfillment, and the emotional burden of guilt and condemnation, reveals the many layers of existential risk. This broader understanding can motivate actions that foster deep resilience and reduce future risks. Emphasizing deep resilience as flexible adaptation at multiple levels such as the individual, family, community, society, and geographical scales in response to the multiple layers of existential threats to individuals, families, communities, societies, geographical regions and the planet itself illustrated how perceptions of existential anxiety may have different motivational effects depending on the level the threat is considered (e.g., individual existential threat versus community threat).

Future research avenues include formalizing the assessment of the existential belief dimensions outlined in Tillich (1952) theory and posited in Table 2 across levels of analysis. For example, directly exploring their relation to existential threat in communities impacted by climate change, communities acting to address climate impacts, and how these dimensions of existential threat relate to policymaker decisions. While instruments for assessing existential anxiety and psychological flexibility have been well researched in individuals (Weems et al., 2004) and systems theory has been the subject of 50 years of research (Shelton, 2025), moving the assessment to other levels of analysis for assessing climate-related issues remains unexplored. However, such an assessment could be used to examine deep resilience in communities that are the most at risk to climate change and other forms of stresses and shocks. For example, in Alaska Native communities, climate change has threatened local infrastructure and access to reliable water. Our work has illustrated that these external issues impact trust in tap water and quality of life overall (Nartey et al., 2025). An assessment of existential sentiments at diverse levels could develop relevant interventions to increase trust in local water sanitation programs, government agencies, and help with the coproduction of policy making in the state. Qualitative and quantitative designs are needed.

We hope that readers of this conceptual analysis will appreciate that existential concerns can be both polarizing and galvanizing. Balancing these concerns to optimal existential action toward deep resilience for individuals, communities, and human society is no easy task in an increasingly polarized world. It will take transdisciplinary action crossing the sciences and arts to fully achieve deep resilience. To that realization and hope we end with these lyrics from the music group Rush “The blacksmith and the artist reflect it in their art. They forge their creativity closer to the heart. Philosophers and ploughmen, each must know their part to sow a new mentality closer to the heart” (Rush, 1977).

## Footnotes

^1^The term environment in this model is used to reflect the “context” as opposed to a biological environment such as a forest per se.

^2^The concept of “geography” in these levels is less commonly noted; however, adding this concept in defining the levels of deep resilience makes particular sense because climate impacts are often differentially salient to those in different geographical contexts. For example, residents in the mid-west United States are less likely to be confronted with the existential threat of climate impacts as residents of Tuvalu. However, it is important to note that these are not just national differences as those in the south Florida in the Keys for example might be more similar to these in Tuvalu in terms of the salience of existential threat. The idea here is that the broad geography of continents and oceans as well as differential climates and structural features are also involved in this broader level and not just nations or specific societies.
